# Characterization of *Campylobacter* associated gastric enteritis among patients with Human Immunodeficiency Virus (HIV) in a hospital in Accra, Ghana

**DOI:** 10.1371/journal.pone.0240242

**Published:** 2020-10-15

**Authors:** Akua Obeng Forson, David Nana Adjei, Michael Olu-Taiwo, Marjorie Ntiwaa Quarchie, Harry Richard Asmah

**Affiliations:** 1 Department of Medical Laboratory Science, School of Biomedical and Allied Health Sciences, College of Health Sciences, University of Ghana, Legon, Ghana; 2 University of Health and Allied Sciences, Accra, Ghana; Nitte University, INDIA

## Abstract

**Background:**

*Campylobacter* infections in HIV positive patients often present with substantial mortality and morbidity when compared to HIV negative patients.

**Aim:**

This study assessed the prevalence of *Campylobacter*, antibiotic resistance phenotypes and genetic factors, and risk of *Campylobacter* infection associated with living in close proximity to domestic animals in HIV patients with gastric enteritis at Korle-Bu Teaching Hospital, Accra, Ghana.

**Methods:**

Resistance to different antibiotics was assessed with Kirby–Bauer disk diffusion method. In addition, all the *Campylobacter* isolates were tested for ampicillin (*bla*_OXA-61_), erythromycin (*aph*-3-1), tetracycline *tet*(O), streptomycin (*aad*E), and the energy-dependent multi-drug efflux pump (*cme*B) resistance genes using multiplex polymerase chain reaction.

**Results:**

Out of a total of 140 (97 females and 43 males) tested patients, 71 (50.7%) patients were positive for *Campylobacter coli*. Female patients aged within 31–40 years (31.6%) and 41–50 years (31.6%) had high frequency of *Campylobacter* infection. Most of the infected patients lived in close proximity to chickens (53.5%), however, some patients (14.1%) lived in close proximity to goats. Phenotypic resistance evaluation revealed widespread resistance to ampicillin (100%), tetracycline (100%), ciprofloxacin (71.8%), erythromycin (69%), and gentamicin (49.3%). However, limited no of isolates contained *bla*_OXA-61_ (1.41%), *cme*B (7.0%) and *tet*(O (7.0%) resistance genes.

**Conclusion:**

HIV patients with gastric enteritis were infected with resistant *Campylobacter coli*. Further studies are required to examine correlation of infected patients with *C*. *coli* and risk of living in close proximity to poultry birds. There is the need for routine investigation of *Campylobacter* in patients with gastroenteritis in order to assist in the development of strategies for combating diseases involving resistant zoonotic bacteria strains.

## Introduction

*Campylobacter* spp. are zoonotic pathogens and a common cause of food-borne infections [1]. Risk factors associated with acquiring *Campylobacter* includes the ingestation of raw milk, partially-cooked poultry or pork meat, raw shellfish and contaminated water [2, 3]. Infections with *Campylobacter* often leads to gastric enteritis and diarrhoea [4]. However, in the last decades, *Campylobacter* spp. are associated with septic arthritis, meningitis, osteomyelitis, neonatal sepsis, bacteraemia and proctocolitis. *Campylobacter* infections occurs in children, immuno-compromised patients and people with multiple comorbidities [5, 6]. Patients with HIV have an increase rate of relapsing *Campylobacter* infections, enteritis, and bacteraemia [7, 8].

Infections with *Campylobacter* spp. can resolve within 3–7 days, however, in severe cases of prolonged enteritis or diarrhoea in immune-suppressed patients’, antibiotic treatments may be required [9, 10]. The fluoroquinolone ciprofloxacin and the macrolides erythromycin, azithromycin and clarithromycin are some of the drugs often recommended for therapy [11], however, high levels of resistance in *Campylobacter* have been reported [12, 13].

The incidence of campylobacteriosis have been found to vary in European countries with notification rate of 45.2 cases per 100,000 inhabitants [14, 15], but in Africa, the prevalence have been found to vary. In Malawi, 85% of children with diarrhoea were reported to be infected with *Campylobacter jejuni* [16], in contrast to studies from Tanzania (9.7%) [8, 17], and Kenya (23%) [18]. Previous studies in Nigeria and Cameroon have reported a 19% and 9.6% *Campylobacter* infection rate [19, 20]. In Ghana, studies with non HIV patients have revealed 17.3% infection rate with high resistance to erythromycin (92.3%) and βeta-lactams (100%) [21]. However, limited information is available on the prevalence of *Campylobacter* associated enteritis or diarrhoea in immune suppressed patients in Ghana. This study aimed to determine the phenotypic and genotypic antibiotic resistance of *Campylobacter* isolates from confirmed HIV infected patients in Ghana with diarrhoea and evaluate the risks of *Campylobacter* infection to patients living in close proximity to some domestic animals.

## Methodology

### Study design

The study was an experimental cross sectional study involving 140 adult confirmed HIV infected in-patients and out-patients with diarrhoea at the Fevers unit of Korle-Bu Teaching Hospital. Korle-Bu Teaching Hospital (KBTH) is one of the largest health care facilities located in Accra and it serves to provide health care for all categories of persons in Ghana [18]. The prevalence of *Campylobacter* enteritis is unknown in Ghana, so an assumed prevalence of 50% was used with the sample size formula for proportions by Berry *et al*., [22] (n = Z^2^ (pq)/E^2^). This provided a minimum sample size of 140 HIV patients. We only included confirmed HIV infected in-patients and out-patients with diarrhoea (watery bowel movement with or without blood stools) attending either continuum of care for antiretroviral therapy refill or any medical condition requiring medical attention at the Fevers unit of Korle-Bu Teaching Hospital from May 2015 –January, 2016. Consenting patients above 18 years only were included in the study. Patients refusing to consent to participate in the study and patients with ongoing antibiotic treatments for gastric enteritis for 1 week were also excluded.

### Isolation of *Campylobacter* spp.

For the isolation of the *Campylobacter* isolates, approximately one gram of each faecal sample was mixed in a sterile container using a sterile swab stick. The excess stool samples were wiped off and the sticks were swabbed onto a (pre-dried) Karmali agar (Oxoid) plate. Plates were incubated at 42°C for 48 hrs in an anaerobic jar with a gas-generating sachet (Oxoid-CampyGen_TM_) to produce micro aerophilic atmospheric conditions to enable the growth of *Campylobacter* spp. [23]. After 48 hrs incubation, smooth, flat, colourless translucent to grey colonies of approximately 1 mm on Karmali agar plates were selected. Since the colonies were often mixed with other bacteria on the Karmali plate, half of the suspected colony was tested for motility using a phase contrast microscope. Colonies showing positive motility were then purified by plating the remaining colonies on 5% horse blood agar plates and Karmali plate. The plates were then incubated for 48 hrs in an anaerobic jar with a gas-generating sachet to produce microaerophilic conditions. Gram-negative, catalase and oxidase positive isolates were stored snap-frozen in 30% glycerol broth at 70°C for further molecular identification (using polymerase chain reaction), antibiotic susceptibility testing and resistance genes determination.

### Antibiotic susceptibility testing

*Campylobacter* isolates were subjected to Kirby–Bauer disc diffusion sensitivity testing per the guidelines of the Clinical and Laboratory Standard Institute (CLSI) with Mueller Hinton Agar supplemented with 5% defibrinated horse blood [24]. The following antibiotics purchased from Thermo Scientific^TM^ Oxoid (United Kingdom) were used: ampicillin, ciprofloxacin, chloramphenicol, erythromycin, streptomycin, gentamicin and tetracycline.

Briefly, on each agar plate, a sterile loop was used to touch two to three morphologically similar colonies of *Campylobacter* spp. and this was inoculate into sterile 0.85% sodium chloride (NaCl) solution until a turbidity of 0.5% McFarland’s standard was obtained. A loopful of the suspension was transferred on to a Mueller-Hinton agar plate with 5% sheep blood, and a sterile cotton swab was used to streak the entire surface of the plate. The lid of the agar plate was left ajar for 3–5 mins to allow excess surface moisture to be absorbed before the application of drug impregnated disks. The agar plates were incubated at 42°C for 24 hrs in an anaerobic jar with a gas-generating sachet to produce microaerophilic conditions. After incubation, zone diameters around the antibiotic discs was measured and classified as sensitive or resistant based on the CLSI [24] and EUCAST [25] break points (Table 1). The reference strains used for quality control of antibiotic discs were *Campylobacter jejuni* ATCC 33560, *Enterococcus*, *faecalis* ATCC 29212, *Escherichia coli* ATCC 25922, and *Staphylococcus aureus* ATCC 29213.

**Table 1:** Guidelines for interpreting antimicrobial susceptibility results for *Campylobacter* spp.

		Zone diameter breakpoint(mm)			
Antibiotic	Disk Content μg	Susceptibility ≥	Resistance <	Reference	
Ampicillin	10	14	14	EUCAST break point for *Enterobacteriacae* [25]	
Ciprofloxacin	5	26	26	EUCAST break point for *C coli* and *C*. *jejuni* [25]	
Erythromycin	15	24	24	EUCAST break point for *C*. *coli* [25]	
Tetracycline	30	30	30	EUCAST break point for *C*. *coli* and *C*. *jejuni* [25]	
Gentamicin	10	17	14	EUCAST break point for *Enterobacteriacae* [25]	
Chloramphenicol	30	17	17	EUCAST break point for *Enterobacteriacae* [25]	
Streptomycin	10	15	12	CLSI break point [24]	

### DNA extractions

DNA extraction was carried out using the Modification of “CTAB method”, in Current Protocols in Molecular Biology (1997) 2.4.1–2.4.5 by John Wiley & Sons, Inc.

Extractions of DNA was carried out by first preparing a cell lysis buffer with adding 50 mL of 10% SDS (sodium dodecyl sulphate), 10 ml of 5M NaCl (sodium chloride), 100ml of 0.5M of EDTA (Ethylenediaminetetraacetic acid), 25 ml of 0.5M Tris (pH = 8) to 315 ml of deionized water. The mixture was autoclaved and left to cool to room temperature. After the preparation, 450 μl of cell lysis buffer was dispensed into a 2 ml sterile eppendorf tubes containing harvested *Campylobacter* spp. cells and 15 μl of proteinase K (20mg/ml) was added. This was briefly mixed by inverting the tube gently for 30 secs and the mixture was incubated at 56°C for 6 hrs. Then 200 μl of 5M NaCl was added and inverted for 15 secs and the mixtures were allowed to stand for 5 mins at room temperature before being centrifuged at 12,000 g for 10 mins. The supernatant was collected into a sterile 2ml eppendorf tube and the sediments were discarded. Cold absolute isopropanol (800 μl) was added to the supernatant and the mixture was inverted for 30 secs before being frozen at -20°C for 30 mins and then re-centrifuged at 12,000 g for 15 mins. The supernatant was discarded and 400 μl of 70% of ethanol was used to wash the DNA pellet collected. This was centrifuged at 12,000 g for 10 mins and the supernatant was discarded. The tube containing the pellet was left opened to allow the pellet to dry up for 1 hr. before 100 μl of nuclease free water was added to the DNA pellet. The mixtures were allowed to sit on the bench for 6 hrs before being used in PCR analysis. DNA was finally stored at -20°C for further use.

### Species identification

The *Campylobacter* species identification was first performed using conventional methods that included the catalase, oxidase and hippurate hydrolysis tests [23]. PCR was then used to confirm species identifications using primers purchased from Integrated DNA Technologies, Inc., USA (https://www.idtdna.com) for the *Campylobacter* genus specific 23S rRNA gene and species specific regions of *Campylobacter jejuni*, *C*. *coli*, *C*. *lari* and *C*. *upsaliensis* (Table 2). In each PCR reaction tube, a mixture of 25 μl contained 12.5 μl of NEB one taq 2X master mix buffer, 0.5 μl of 10 μM of each oligonucleotide primers, 2 μl DNA template and 9.5μl of nuclease free water [26]. Cycling condition were set at 95°C for 5 mins, 30 cycles of denaturation at 95°C for 30 secs., annealing at 59°C for 30secs., extension at 68°C for 2 mins. and ending with a final extension at 68°C for 10 mins. The PCR products were then electrophoresed on a 2% agarose gel stained with ethidium bromide.

**Table 2:** Primers used for PCR in this study.

	Primer Sequence (5’-3’)		Amplicon	Annealing	
Primer	Forward	Reverse	Sizes (bp)	Temperatures	Reference
CJF	acttctttattgcttgctgc	gccacaacaagtaaagaagc	323	59°C	[26]
CCF	gtaaaaccaaagcttatcgtg	tccagcaatgtgtgcaatg	126	59°C	[26]
CLF	tagagagatagcaaaagaga	tacacataataatcccaccc	251	59°C	[26]
CUF	aattgaaactcttgctatcc	tcatacattttacccgagct	204	59°C	[26]
CFF	gcaaatataaatgtaagcggagag	Tgcagcggccccacctat	435	59°C	[26]
23SF	tataccggtaaggagtgctggag	atcaattaaccttcgagcaccg	650	59°C	[26]
*tet*(O)	gcgttttgtttatgtgcg	atggacaacccgacagaag	559	49°C	[27]
*cme*B	tcctagcagcacaatatg	Agcttcgatagctgcatc	241	49°C	[28]
*bla*_OXA-61_	agagtataatacaagcg	Tagtgagttgtcaagcc	372	49°C	[28]
*aphA-3-1*	tgcgtaaaagatacggaag	Caatcaggcttgatcccc	701	49°C	[28]
*aadE*1	gaacaggatgaacgtattcg	Gcatatgtgctatccagg	837	49°C	[29]

### Molecular analysis of *Campylobacter* for resistant genes

Resistance genes investigations were carried out in all the isolates. The genomic DNA extracted above for the *Campylobacter* isolates were tested for *tet*(O), *aph*-3-1, *cme*B and *bla*_OXA-61_ genes using PCR. In each PCR reaction tube, a mixture of 25 μl contained 12.5 μl of NEB onetaq 2X master mix buffer, 0.1 μl of 10 μM of each of the resistant gene primer (Table 2), 2 μl DNA template and 9.5 μl of nuclease free water was used [26].

Cycling condition was set at 95°C for 5 mins, 35 cycles of denaturation at 95°C for 30secs, annealing at 49°C for 45 secs, extension at 68°C for 2 mins and ending with a final extension at 68°C for 10 mins. The PCR products were then electrophoresed on a 2% agarose gel stained with ethidium bromide. The sizes of the various amplicons was determined by comparison with 100 bp ladder. Some of the amplified DNA generated with positive specific primers were sequenced in order to confirm the identity of resistance genes detected.

### Data handling and statistical analysis

The data generated were entered into Microsoft Excel and analyzed using GraphPad Prism software, version 6. In all cases, p-values less than 0.05 were considered statistically significant. Initially, the association between each exposure and the presence of infection was assessed using the Pearson’s Chi-squared test. Odds ratios were then computed to measure the direction and strength of association. Prevalence was calculated by dividing the number of occurrences of *Campylobacter* species in the HIV infected patients by the total number of tested HIV infected patients.

### Ethics approval and consent

The study was approved by the Noguchi Memorial Institute for Medical Research Institution Review Board, University of Ghana, Legon (Ethics approval reference Number: NMIMR-IRB CPN 035/14-15). Participation was voluntary and written consent from patients was taken in accordance with the ethical committee’s guidelines. Patients attending the hospital on clinic days were only included in this study after a signed written informed consent was obtained.

## Results

### Prevalence of *Campylobacter* isolates in male and female patients

Out of a total of 140 (97 females and 43 males) tested consenting patients, 71 (50.7%) patients were infected with *Campylobacter* spp. (Table 3). Sixty one percent of females and 38.7% of the males were infected with *Campylobacter*. Female patients aged within 31–40 years (31.6%) and 41–50 years (31.6%) were the age groups mostly infected. Whilst males aged between 41–50 years (64.3%) and 51–60 years (28.6%) were predominantly infected, lower prevalence was found for both male and female patients within 20–30 and 71–80 years respectively (Table 3). The difference in rate of infection in the males and females was not significant (P < 0·05).

**Table 3:** Distribution of ages and *Campylobacter coli* infections in patients.

**Females**				
**Ages**	**Total No. of Tested female patients**	*Campylobacter*		
	**(n = 97)**	Positive (n = 57, %)	Odds ratio	P-value
20–30	8	5 (8.8)		
31–40	35	18 (31.6)		
41–50	30	18 (31.6)	1.583	0.0991
51–60	18	12 (21.1)		
61–70	4	3 (5.3)		
71–80	2	1 (1.8)		
**Males**				
**Ages**	**Total No. of tested male patients**	*Campylobacter*		
	**(n = 43)**	Positive (n = 14, %)	Odds ratio	P-value
20–30	2	0		
31–40	6	0		
41–50	14	9(64.3)	1.589	0.3758
51–60	12	4(28.6)		
61–70	5	1(7.2)		
71–80	4	0		

### Risk factor of domestic animals and *Campylobacter* infection

Seventy three percent (52/71) of the infected patients reported to live in close proximity to domestic animals (Table 4). Most of the positive patients had contacts with chickens [53% (38/71)] and dogs [14.1% (10/71)]. A few of the *Campylobacter* culture negative patients also lived in close proximity to chickens [31.9% (22/69)] and dogs [11.6% (8/69)]. The difference in *Campylobacter* positive patients and negative patients living in proximity to domestic animals was not statistically significant (P < 0·05).

**Table 4:** Distribution of ages of patients and contact with domestic animals patients.

	Positive Patients (n = 71)				Negative Patients (n = 69)			
	Domestic Animals				Domestic Animals			
Ages	Cats	Dogs	chickens	Goats	Cats	Dogs	chickens	Goats
20–30	0	0	2	1	0	0	0	1
31–40	0	1	12	3	0	1	9	2
41–50	1	2	10	5	0	1	7	3
51–60	0	0	10	0	1	1	6	2
61–70	0	0	3	1	0	0	0	0
71–80	0	0	1	0	0	0	0	0
**Total (%)**	**1 (1.4)**	**3 (4.2)**	**38 (53.5)**	**10 (14.1)**	1 (1.4)	3 (4.3)	22 (31.9)	8 (11.6)

### Prevalence of *Campylobacter* spp.

*Campylobacter coli* [50.7% (71/140)] was the predominate species isolated. None of the *Campylobacter* isolates in this study produced any species-specific band for *Campylobacter upsaliensis*, *Campylobacter jejuni* and *Campylobacter lari*.

### Antibiotic resistance and resistance genes profiles of *Campylobacter* isolates

All the *Campylobacter* isolates were resistant to ampicillin (100%) and tetracycline (100%) (Table 5). Whilst resistance to chloramphenicol, erythromycin, gentamicin and ciprofloxacin were 28.2%, 69%, 49.3% and 71.8% respectively.

**Table 5:** Prevalence of phenotypic antibiotic resistance and resistance genes in *Campylobacter coli* isolates.

**Antibiotic Tested by disc diffusion**	**No. of Phenotypic resistant *C*. *coli* (%)**
**Resistant genes tested**	No. of isolates positive for resistance genes
**Ampicillin**	71 (100)
***bla*_OXA-61_**	1 (1.41)
**Erythromycin**	49 (69.0)
**Ciprofloxacin**	51 (71.8)
***cme*B**	5 (7.0)
**Tetracycline**	71 (100)
***tet*(O)**	6 (8.4)
**Streptomycin**	45 (63.4)
***aad*E**	4 (5.6)
**Gentamicin**	35(49.3)
**chloramphenicol**	20 (28.2)

All the *Campylobacter coli* isolates were tested for *tet*(O), *aph*-3-1, *cme*B and *bla*_OXA-61_ antibiotic resistance genes. This study revealed a few (7%) of the *C*. *coli* isolates resistant to ciprofloxacin contained the *cme*B gene (Table 5). Though high (100%) phenotypic resistance to tetracycline was detected, only six (8.4%) *Campylobacter* isolates was positive for the *tet*(O) gene. In addition, *bla*_OXA-61_ gene was present in only one ampicillin resistant isolate, and *aad*E gene was present in four streptomycin resistant isolates.

## Discussion

*Campylobacter* spp. have been identified as the etiologic agent in sporadic cases of gastroenteritis and gastrointestinal infection in both developed and developing counties [30]. Even though this hospital-based study with 140 HIV positive patients within the ages of 20–80 years with watery diarrhea is small to make any statistical significant information on seasonal (raining or dry season) distribution, however, this is the first study to evaluate the prevalence of *Campylobacter* spp. infections in HIV infected patients in Ghana and the potential risk of patients living in close proximity to domestic animals.

### The prevalence of *Campylobacter* infection

The overall prevalence of *Campylobacter* infection in the tested patients with diarrhoea was 50.7%. This prevalence is higher than previous prevalence reported in Germany (18.6%) [31], Southern Ireland (4.7%) [32], Kenya (12.7%) [33], and Malawi (14%) [16]. The differences in prevalence in the different countries may be associated with varying seasonal differences, sensitivity of detection methodologies, scope of the case profile studied, as well as differences in the standard and stringency of biocontrol protocols, surveillance bias, food practices, and climatic conditions [15].

The predominant *Campylobacter* spp. Identified in this study was *C*. *coli*, which is the strain responsible for 25% of all gastroenteritis cases caused by *Campylobacter* species [10]. Findings in the study are in contrast to studies from USA, Paris, and South Africa which reported a higher prevalence of *C*. *jejuni* than *C*. *coli* [34–36]. The high prevalence of *C*. *coli* detected in this study may suggest that faeces of infected domesticated animals is enabling the spread of *C*. *coli* in the environment and thus allowing contamination of the patients [37]. This may also be because in most developing countries, exposure to *Campylobacter* infections are common in early life, leading to development of protective immunity in children and continuous asymptomatic excretion that can be a potential risk to immune compromised patients [38, 39].

### Antibiotic resistance and resistance genes

Over the years, antibiotic resistance in *Campylobacter* spp. in humans and animals has become a public health concern in both developed and developing countries [13], however information on *Campylobacter* in HIV patients are limited. *Campylobacter* infections are self-limiting, and antibiotics such as macrolides (erythromycin), fluoroquinolones (ciprofloxacin) and tetracycline may be recommended in severe cases or in immunocompromised patients [40]. However, in this study most of the *Campylobacter coli* isolates were resistant to tetracycline (100%), ciprofloxacin (71.8%) and erythromycin (69%). In contrast to this study, a previous study reported lower resistance for tetracycline (55%), erythromycin (38.9%) and ciprofloxacin (33.3%) in *C*. *coli* isolates from patients with dysentery [34] in South Africa. Whilst a similar study in Kenya reported varying resistance to tetracycline (57.1%), ciprofloxacin (28.9) and erythromycin (85.1%) [40], in India, a study from 2010 to 2012 reported an increase in resistance to ciprofloxacin and erythromycin from 71.4% to 86.1% and 6.1% to 22.2% respectively in children [41]. The varying prevalence observed in the different countries may be associated with varying levels of dependency on fluoroquinolones, macrolides and tetracyclines in clinical practice and possible utilization of some of the antibiotics in veterinary practice or animal husbandry [1, 2].

In this study, all the *Campylobacter* isolates were resistant to tetracycline, but only six of the resistant isolates contained the *tet*(O) gene. In contrast to this study, a previous study by Shobo *et al*., [34] in South Africa reported *tet*(O) gene in all tetracycline resistant *C*. *coli* and *C*. *jejuni* isolates. Similarly, a previous study from a different region in Ghana (Kumasi) reported a 92% tetracycline resistance to *C*. *coli* isolates in patients with enteritis, however the study did not evaluate the prevalence of *tet*(O) gene. Tetracycline resistance is primarily mediated by the ribosomal protection protein (*tet*O) that are located on a self-transmissible plasmid or in the chromosome when it is not self-mobile [42]. The low prevalence of *tet*(O) gene in the tetracycline resistant isolates in this study may be associated with the contribution of efflux pumps for tetracycline resistant isolates which are not associated with the expression of *tet*(O) gene [43]. The multidrug efflux gene c*me*B which is able to confer intrinsic resistance to fluoroquinolones and macrolide was present in a few of the resistant isolates [44]. The lower prevalence of *cme*B in the *C*. *coli* strains might indicate a higher sequence variation in the *cme*B gene in the tested isolates, and probably the involvement of other periplasmic fusion protein (*Cme*ABC) genes with the intrinsic resistance in the *Campylobacter* isolates [45, 46]. Other putative efflux pumps including *Cme*DEF and *Cme*G, [46–48] and antibiotic exclusion via the major outer membrane porin (MOMP) [49], lipooligosaccharide and capsule [50] can also contribute to intrinsic resistance. The role of *Cme*ACDEFG genes in our isolates needs to be further studied to determine other specific target pathways for antimicrobial resistance.

None of erythromycin-resistant isolates harboured the *aph*-3-1 gene, however, four streptomycin resistant *C*. *coli* isolates contained the *aad*E gene. Resistance to the above antibiotics is generally due to inactivation of the drugs by aminoglycoside phosphotransferases (APH) or adenylyltransferases (AAD) [3], the presence *aadE* in *C*. *coli* constitutes further evidence for the occurrence of genetic transfer of information from gram positive to gram-negative bacteria under natural conditions [51].

### Domestic animals and risk factors for *Campylobacter* infections

The consumption of raw milk, pork or poultry products and contact with animals are a significant risk factor for *Campylobacter* enteritis [3, 52]. Out of the 71 positive *Campylobacter* patients, 73% reported to live in close proximity domestic animals in contrast to the negative patients (49%). Most of the patients however, reported to live in close proximity to poultry birds or were engaged in poultry husbandry. *Campylobacter* spp. (primarily *C*. *jejuni* and *C*. *coli*) are commensals in birds and commonly associated with enteritis in domesticated animals and humans [53]. Other species such as *C*. *fetus*, *C*. *helveticus*, *C*. *hyointestinalis*, *C*. *lari*, *C*. *upsaliensis*, *C*. *sputorum*, and *C*. *ureolyticus* can infect a wide range of food animals such as poultry, cattle, pigs, sheep and ostriches; and in pets, including cats and dogs [54, 55]. Findings from this study are in conformity to Potter *et al*., [52] study in the United States which reported that persons living or engaged in poultry husbandry are at a higher risk for *Campylobacter* infections. We did not collect information on the types of antibiotics used in the feeds or for treatment of the domesticated chickens. This was due to the relatively small number of patients who were willing to enrol in the study did not have sufficient information to enable the study evaluate such information or the association of patients infections with rural or urban location.

Nevertheless, reported incidence and prevalence of *Campylobacter* infections among persons infected with human immunodeficiency virus (HIV) occur at a higher rate than what is observed in the general population [56, 57]. Close monitoring and differential diagnosis of *Campylobacter* in HIV patients with persistent diarrhoea is required even if the organism is not identified in stools [59]. This is because the severity and duration of *Campylobacter* infections are more likely to be increased among HIV-infected persons [58, 59].

## Conclusion

This study revealed that HIV patients with gastric enteritis were positive for *Campylobacter coli*. Females who were often infected with *C*. *coli* were found to live in close proximity to poultry birds. The isolated *C*. *coli* strains were resistant to tetracycline, ciprofloxacin and erythromycin, and a few of the resistant *C*. *coli* isolates harboured the tested resistance genes.

Findings from this study emphases the need for the empowerment of diagnostic laboratories in West Africa, especially Ghana, to detect emerging *Campylobacter* species and the use of whole genome sequencing to evaluate the correlation between resistance phenotypes and genotypes in order to understand and assist the development of new strategies for combating antimicrobial resistance, including improving foodborne disease surveillance programs involving bacteria of zoonotic importance like *Campylobacter*.

### Strength and weakness

Aside the phenotypic testing of antibiotic resistance in *Campylobacter* isolates in HIV patients, the use of PCR to test for resistance genes gave power to the study. However, the study was limited in accessing the medical history of the patients, hence, our inability to link our findings to other clinical conditions. We could also not determine the virulence factors of the culture positive isolates, to confirm the colonization capacity of the bacteria to the host's intestinal cells. A bigger multi-regional survey involving HIV and non-HIV patients which will incorporate treatment and other variables is planned pending appropriate funding. Also, the relatively small sample size of this study may have failed to detect any *C*. *jejuni* among the HIV patients.

## Supporting information

S1 Appendix Results–Campylobacter speciation and resistance genes. (DOCX)
