# Supplementary material for: Characterization of Campylobacter associated gastric enteritis among patients with Human Immunodeficiency Virus (HIV) in a hospital in Accra, Ghana
Source: PLoS One. 2020 Oct 15;15(10):e0240242. doi: 10.1371/journal.pone.0240242 (PMC7561167; doi:10.1371/journal.pone.0240242)
Supplement: S1 Appendix — (DOCX) [file pone.0240242.s001.docx]

**S1 Appendix. Results – Campylobacter speciation and resistance genes.**

| **SAMPLE ID** | **Sex** | ***tet*(O)** | ***cme*B** | **bla_OXA-61_** | ***aphA-3-1*** | ***aad*E** | **23S rRNA** | ***C. coli*** | ***C. jejuni*** | ***C. lari*** | ***C. upsaliensis*** |
| --- | --- | --- | --- | --- | --- | --- | --- | --- | --- | --- | --- |
| 54 | Female | Negative | Negative | Negative | Negative | Negative | Positive | Positive | Negative | Negative | Negative |
| 52 | Male | Negative | Negative | Negative | Negative | Negative | Positive | Positive | Negative | Negative | Negative |
| 33 | Female | Negative | Negative | Negative | Negative | Negative | Positive | Positive | Negative | Negative | Negative |
| 6 | Female | Negative | Negative | Negative | Negative | Negative | Positive | Positive | Negative | Negative | Negative |
| 19 | Male | Negative | Negative | Negative | Negative | Negative | Positive | Positive | Negative | Negative | Negative |
| 43 | Female | Negative | Negative | Negative | Negative | Negative | Positive | Positive | Negative | Negative | Negative |
| 58 | Female | Negative | Negative | Negative | Negative | Negative | Positive | Positive | Negative | Negative | Negative |
| 22 | Female | Negative | Positive | Negative | Negative | Negative | Positive | Positive | Negative | Negative | Negative |
| 59 | Female | Negative | Negative | Negative | Negative | Negative | Positive | Positive | Negative | Negative | Negative |
| 18 | Female | Negative | Negative | Negative | Negative | Negative | Positive | Positive | Negative | Negative | Negative |
| 64 | Female | Negative | Negative | Negative | Negative | Negative | Positive | Positive | Negative | Negative | Negative |
| 44 | Female | Negative | Positive | Negative | Negative | Negative | Positive | Positive | Negative | Negative | Negative |
| 24 | Female | Negative | Negative | Negative | Negative | Negative | Positive | Positive | Negative | Negative | Negative |
| 2 | Female | Negative | Negative | Negative | Negative | Negative | Positive | Positive | Negative | Negative | Negative |
| 47 | Female | Positive | Positive | Negative | Negative | Negative | Positive | Positive | Negative | Negative | Negative |
| 53 | Female | Negative | Negative | Negative | Negative | Negative | Positive | Positive | Negative | Negative | Negative |
| 4 | Female | Negative | Negative | Negative | Negative | Negative | Positive | Positive | Negative | Negative | Negative |
| 44 | Female | Negative | Negative | Negative | Negative | Negative | Positive | Positive | Negative | Negative | Negative |
| 92 | Female | Negative | Negative | Negative | Negative | Negative | Positive | Positive | Negative | Negative | Negative |
| 80 | Female | Negative | Negative | Negative | Negative | Negative | Positive | Positive | Negative | Negative | Negative |
| 106 | Male | Negative | Negative | Negative | Negative | Negative | Positive | Positive | Negative | Negative | Negative |
| 63 | Female | Negative | Negative | Negative | Negative | Negative | Positive | Positive | Negative | Negative | Negative |
| 95 | Male | Negative | Negative | Negative | Negative | Negative | Positive | Positive | Negative | Negative | Negative |
| 76 | Male | Negative | Negative | Negative | Negative | Negative | Positive | Positive | Negative | Negative | Negative |
| 79 | Female | Negative | Negative | Negative | Negative | Negative | Positive | Positive | Negative | Negative | Negative |
| 87 | Male | Negative | Negative | Negative | Negative | Negative | Positive | Positive | Negative | Negative | Negative |
| 50 | Male | Negative | Negative | Negative | Negative | Negative | Positive | Positive | Negative | Negative | Negative |
| 66 | Female | Negative | Negative | Negative | Negative | Negative | Positive | Positive | Negative | Negative | Negative |
| 16 | Female | Negative | Negative | Negative | Negative | Negative | Positive | Positive | Negative | Negative | Negative |
| 39 | Female | Negative | Negative | Negative | Negative | Negative | Positive | Positive | Negative | Negative | Negative |
| 34 | Female | Negative | Negative | Negative | Negative | Negative | Positive | Positive | Negative | Negative | Negative |
| 78 | Female | Negative | Negative | Negative | Negative | Negative | Positive | Positive | Negative | Negative | Negative |
| 72 | Female | Negative | Negative | Negative | Negative | Negative | Positive | Positive | Negative | Negative | Negative |
| 68 | Female | Negative | Negative | Negative | Negative | Negative | Positive | Positive | Negative | Negative | Negative |
| 100 | Male | Negative | Negative | Negative | Negative | Negative | Positive | Positive | Negative | Negative | Negative |
| 37 | Female | Negative | Negative | Negative | Negative | Negative | Positive | Positive | Negative | Negative | Negative |
| 7 | Male | Negative | Negative | Negative | Negative | Negative | Positive | Positive | Negative | Negative | Negative |
| 45 | Female | Negative | Negative | Negative | Negative | Negative | Positive | Positive | Negative | Negative | Negative |
| 93 | Female | Negative | Positive | Negative | Negative | Positive | Positive | Positive | Negative | Negative | Negative |
| 38 | Female | Negative | Negative | Negative | Negative | Negative | Positive | Positive | Negative | Negative | Negative |
| 5 | Female | Negative | Negative | Negative | Negative | Negative | Positive | Positive | Negative | Negative | Negative |
| 30 | Female | Positive | Negative | Negative | Negative | Positive | Positive | Positive | Negative | Negative | Negative |
| 77 | Female | Positive | Negative | Negative | Negative | Positive | Positive | Positive | Negative | Negative | Negative |
| 51 | Male | Positive | Negative | Negative | Negative | Negative | Positive | Positive | Negative | Negative | Negative |
| 29 | Female | Negative | Positive | Negative | Negative | Negative | Positive | Positive | Negative | Negative | Negative |
| 28 | Female | Negative | Negative | Negative | Negative | Negative | Positive | Positive | Negative | Negative | Negative |
| 88 | Male | Negative | Negative | Negative | Negative | Negative | Positive | Positive | Negative | Negative | Negative |
| 1 | Female | Negative | Negative | Negative | Negative | Negative | Positive | Positive | Negative | Negative | Negative |
| 35 | Male | Negative | Negative | Negative | Negative | Negative | Positive | Positive | Negative | Negative | Negative |
| 61 | Male | Positive | Negative | Negative | Negative | Negative | Positive | Positive | Negative | Negative | Negative |
| 85 | Female | Positive | Negative | Positive | Negative | Negative | Positive | Positive | Negative | Negative | Negative |
| 82 | Female | Negative | Negative | Negative | Negative | Negative | Positive | Positive | Negative | Negative | Negative |
| 27 | Female | Negative | Negative | Negative | Negative | Negative | Positive | Positive | Negative | Negative | Negative |
| 99 | Female | Negative | Negative | Negative | Negative | Positive | Positive | Positive | Negative | Negative | Negative |
| 3 | Female | Negative | Negative | Negative | Negative | Negative | Positive | Positive | Negative | Negative | Negative |
| 57 | Female | Negative | Negative | Negative | Negative | Negative | Positive | Positive | Negative | Negative | Negative |
| 66 | Female | Negative | Negative | Negative | Negative | Negative | Positive | Positive | Negative | Negative | Negative |
| 23 | Female | Negative | Negative | Negative | Negative | Negative | Positive | Positive | Negative | Negative | Negative |
| 13 | Female | Negative | Negative | Negative | Negative | Negative | Positive | Positive | Negative | Negative | Negative |
| 31 | Female | Negative | Negative | Negative | Negative | Negative | Positive | Positive | Negative | Negative | Negative |
| 74 | Female | Negative | Negative | Negative | Negative | Negative | Positive | Positive | Negative | Negative | Negative |
| 67 | Female | Negative | Negative | Negative | Negative | Negative | Positive | Positive | Negative | Negative | Negative |
| 81 | Male | Negative | Negative | Negative | Negative | Negative | Positive | Positive | Negative | Negative | Negative |
| 40 | Female | Negative | Negative | Negative | Negative | Negative | Positive | Positive | Negative | Negative | Negative |
| 62 | Female | Negative | Negative | Negative | Negative | Negative | Positive | Positive | Negative | Negative | Negative |
| 2 | Female | Negative | Negative | Negative | Negative | Negative | Positive | Positive | Negative | Negative | Negative |
| 12 | Female | Negative | Negative | Negative | Negative | Negative | Positive | Positive | Negative | Negative | Negative |
| 4 | Female | Negative | Negative | Negative | Negative | Negative | Positive | Positive | Negative | Negative | Negative |
| 46 | Female | Negative | Negative | Negative | Negative | Negative | Positive | Positive | Negative | Negative | Negative |
| 71 | Female | Negative | Negative | Negative | Negative | Negative | Positive | Positive | Negative | Negative | Negative |
| 73 | Female | Negative | Negative | Negative | Negative | Negative | Positive | Positive | Negative | Negative | Negative |
